# Gastric Intestinal Metaplasia: Demographic and Epidemiological Characterization in Puerto Rican Hispanics (2012-2014)

**DOI:** 10.1155/2021/9806156

**Published:** 2021-02-23

**Authors:** Jorge J. Cruz-Cruz, María González-Pons, Adrián Cora-Morges, Marievelisse Soto-Salgado, Giancarlo Colón, Kimberly Alicea, Kathia Rosado, Douglas R. Morgan, Marcia Cruz-Correa

**Affiliations:** ^1^UPR School of Medicine, San Juan, PR, USA; ^2^UPR Comprehensive Cancer Center, San Juan, PR, USA; ^3^UPR Rio Piedras Campus, San Juan, PR, USA; ^4^VA Caribbean Healthcare System, San Juan, PR, USA; ^5^Division of Gastroenterology and Hepatology, University of Alabama at Birmingham (UAB), AL, USA

## Abstract

**Background and Aims:**

Gastric cancer is the 5^th^ leading cause of cancer mortality worldwide and the leading infection-associated cancer. *Helicobacter pylori* is the most common chronic bacterial infection in humans and the major predisposing factor for the development of gastric intestinal metaplasia (GIM), the principal preneoplastic lesion in the gastric carcinogenesis pathway. GIM surveillance is now recommended for individuals among high-risk subgroups by three major gastroenterology societies in Europe, England, and U.S. Our objective was to provide the initial epidemiologic data for GIM among Hispanics in Puerto Rico.

**Methods:**

Using a cross-sectional study design, we analyzed an extensive pathology database (*n* = 43,993) that captured approximately 50% of all endoscopy biopsies taken during 2012-2014 at academic, public, and private sectors in Puerto Rico. Prevalence estimates of GIM, GIM subgroups, and *H. pylori* status were estimated using logistic regression models.

**Results:**

A total of 4,707 GIM cases were identified during the study period for a prevalence rate of 10.7%. *H. pylori* was detected in 26.9% (95% CI: 25.7-28.2) of the GIM cases. The majority of the pathology reports lacked information regarding the high-risk subtypes (99.6%) and extension (71.2%).

**Conclusions:**

The prevalence of GIM among Hispanics living in Puerto Rico may be higher than in U.S. mainland non-Hispanic populations. The prevalence of *H. pylori* detected in our study population was comparable to the rates reported in the mainland U.S. Standardization of the endoscopy biopsy protocol and pathology reporting is needed to characterize and risk stratify GIM surveillance programs in Puerto Rico.

## 1. Introduction

Worldwide, gastric cancer is the 5^th^ leading cause of cancer mortality and the leading infection-associated cancer [1]. Although gastric cancer is not currently among the top ten most diagnosed cancers or leading causes of cancer death in the mainland U.S., the American Cancer Society projects 27,600 incident cases and approximately 11,010 deaths in 2020. However, in Puerto Rico, gastric cancer continues to be a commonly diagnosed malignancy. It is the 15^th^ most commonly diagnosed cancer on the island and is the 6^th^ and 8^th^ leading cause of cancer death among men and women, respectively [2, 3].

Noncardia gastric cancer, arising from the antrum, incisura, body, and/or fundus, represents the majority of gastric cancers in the U.S. [4]. This histologic subtype of gastric cancer arises through a stepwise progression starting with chronic gastritis followed by atrophic gastritis, gastric intestinal metaplasia (GIM), dysplasia, and adenocarcinoma [5]. GIM, considered an important intermediate preneoplastic lesion, usually develops following *H. pylori*-driven atrophic gastritis and is characterized by the replacement of normal gastric epithelium by intestine-like glandular structures [6].

In East Asia and South America, where gastric cancer incidence is high, the pooled prevalence rates for GIM were 21.0% and 23.9%, respectively [7]. In the mainland U.S., a region with a low gastric cancer burden, multiple large nationwide studies report prevalence rates ranging from 4.8-9.3%, with the highest prevalence of GIM observed among Hispanics compared to other racial/ethnic groups [7–9]. Gastric cancer continues to be one of the leading causes of cancer death in Puerto Rico [2]; however, there is limited information regarding the prevalence of GIM and its risk factors among Hispanics living in Puerto Rico. A better understanding of the risk factors for GIM and gastric cancer among Puerto Rican Hispanics is needed in order to inform the development of risk-stratified screening and surveillance strategies to reduce gastric cancer health disparities among this understudied Hispanic subpopulation.

## 2. Materials and Methods

### 2.1. Study Population

**Figure 1:**
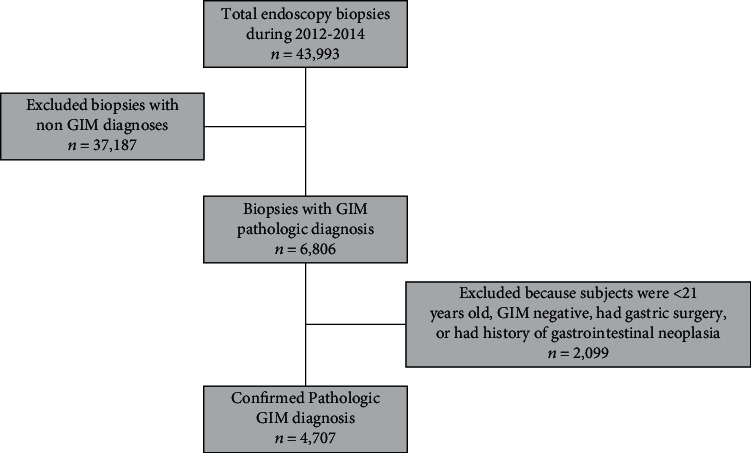
Flow chart of inclusion and exclusion process.

A cross-sectional study design was used to analyze pathology reports obtained from endoscopies with a GIM diagnosis during 2012-2014 from Hato Rey Pathology Associates Inc., a private laboratory that receives samples from over 50% of all practicing gastroenterologists in Puerto Rico. A total of 43,993 endoscopic biopsies were evaluated. All pathologies without a GIM diagnosis (*n* = 1,556), with previous and/or current history of gastrointestinal neoplasia (gastric cancer *n* = 61; gastric polyps *n* = 436) and/or gastric surgery (*n* = 24), or from individuals younger than 21 years of age were excluded (*n* = 22) (Figure 1). All patients who underwent endoscopies and had a pathological diagnosis of GIM during the study period were included (*n* = 6,806). The unidentified pathology reports provided had information on patient age, gender, city of residence, presence of GIM, pathological description of GIM, location of gastric biopsies, and presence of *H. pylori* infection, gastric ulcers, and/or gastritis. This study was approved by the University of Puerto Rico Medical Sciences Campus Institutional Review Board (Protocol # A2210615).

### 2.2. Statistical Analyses

Characteristics were described using frequency distributions for categorical variables and summary measures for quantitative variables. Prevalence estimates of GIM, overall and by histological subtype (complete vs. incomplete), were calculated. Prevalence estimates of *H. pylori* infection, overall and by demographic characteristics, were also calculated. Statistical analyses were performed using STATA 12.0 (STATA Corp.).

## 3. Results

### 3.1. Prevalence of GIM

A total of 4,707 pathology reports with a GIM diagnosis were included in the study, with a mean prevalence of 10.7% during 2012-1014 (Table 1). The prevalence of GIM per year was 10.2%, 10.7%, and 11.3% for 2012, 2013, and 2014, respectively. The demographic and clinicopathological characteristics for our study population are shown in Table 1. GIM was more frequently identified in females compared to males. The mean age at GIM diagnosis was 66.1 ± 12.3 years; median age at diagnosis was 67 years. As expected, the prevalence of GIM was higher in the older age groups, with the highest prevalence among patients ≥ 65 years old. Information on GIM extension (limited vs. extensive) and histologic type (complete vs. incomplete) was not available in most cases; 99.6% and 71.2% of the pathology reports did not specify the histological type and extent of the disease, respectively.

The distribution of biopsy locations according to GIM status is presented in Table 2. A total of 7,253 biopsies were taken in 4,707 endoscopies. Most biopsies were taken from the antrum (60.6%); only 1.5% were taken from the incisura angularis. The prevalence of GIM was highest among biopsies taken from the incisura angularis (92.7%) and the antrum (91.3%). Adherence with the Sydney protocol for endoscopic screening for GIM was documented in only 1.5% of the endoscopies included in this study.

### 3.2. Prevalence of *H. pylori* Infection

In our study population, the prevalence of active *H. pylori* infections in subjects with GIM was 26.9% (Table 3). A higher number of *H. pylori* infections were detected in male subjects with GIM (31.5%) and among older subjects, where the highest prevalence was observed among patients 50-64 years old (30.3%).

## 4. Discussion

Gastric cancer health disparities have been reported among racial/ethnic minorities in U.S., including Puerto Rican Hispanics [2, 3, 10]. GIM is considered an important intermediate preneoplastic lesion in the gastric carcinogenesis pathway, and individuals with GIM have been reported to have an increased risk of gastric cancer [7]. We report the epidemiologic data for GIM among Hispanics living in Puerto Rico for the first time, a Hispanic subpopulation with high gastric burden [2, 3].

The overall prevalence of GIM in our Puerto Rican Hispanic cohort was 10.7%, which is higher to what has been reported in the U.S. (4.8%) using pooled data, but lower compared to mainland U.S. Hispanics (23.3%) [7]. However, in studies evaluating GIM across the U.S., the overall prevalence of GIM ranged from 7.6 to 9.3%. Prevalence among Hispanics ranged from 12 to 13.5%, which is comparable to the prevalence of GIM observed in our cohort [8, 9]. Our analysis also showed higher frequencies of GIM among older age subgroups, which has been previously reported and parallels the observed increase in *H. pylori* seroprevalence observed among older individuals in Puerto Rico [11, 12]. Future studies are needed to identify additional risk factors that contribute to higher prevalence rates of GIM among Hispanics living in Puerto Rico, despite the fact that *H. pylori* seroprevalence rates in Puerto Rico (33%) were comparable to rates reported in the mainland U.S. (30.7%) [11, 13].

A higher prevalence of GIM was observed among women compared to men in our cohort, which is in contrast with other studies where a higher number of GIM cases were diagnosed among men [14, 15]. Possible explanations for the higher prevalence of GIM among Puerto Rican women could be related to GIM in the setting of autoimmune atrophic gastritis (a known etiological factor for GIM that most commonly affects females) and/or the fact that women seek more health care compared to men [16, 17]. The observed gender difference in the prevalence GIM may suggest an underestimation the true prevalence of GIM among Puerto Rican Hispanics and warrants further investigation taking into account detailed histological information, such as oxyntic prevalence of GIM, to try to understand the factors contributing to a higher number of GIM cases among women in Puerto Rico.

One of the most striking findings in our study was the lack of information in pathology reports regarding the histological type and extension of GIM. Current clinical practice guidelines for the management of GIM define patients with a higher risk of gastric cancer as those with incomplete GIM, extensive GIM, and/or family history of gastric cancer [18–20]. These guidelines recommend that biopsies be taken according to the updated Sydney, operative link on gastritis assessment (OLGA), and operative link on gastritis assessment using IM (OLGIM) systems for the cancer risk assessment (two biopsies from the antrum, two from the body, and one from the incisura) [21–23]. The distribution of the biopsy locations during our study period (Table 2) suggests that the majority of the endoscopies performed did not follow biopsy guidelines, which may also explain why there was limited pathologic information on GIM extension. Our findings support that current endoscopic sampling in Puerto Rico should be standardized in order to properly diagnose GIM and to increase the detection of high-risk GIM subtypes, thereby identifying individuals at a higher risk of gastric cancer that need tailoring of their endoscopic surveillance.

The highest prevalence of GIM was observed in the incisura angularis (Table 3), which is the most likely site to reveal gastric preneoplastic lesions, and is recommended to be examined in combination with biopsies from antrum and corpus in the updated Sydney system and OLGA and OLGIM systems [21–24]. Interestingly, 45.5% of biopsies taken in the cardia were positive for GIM, a site not included in the current biopsy sampling recommendations [21–23]. These lesions may have developed as a result of *H. pylori*-independent risk factors, such obesity and gastroesophageal reflux disease, and warrant future studies [24].

In our study population, 26.9% of individuals with GIM were positive for *H. pylori.* Interestingly, we observed a higher prevalence of *H. pylori*-positive GIM cases among males compared to females, which is consistent with the higher number of males diagnosed with gastric cancer in Puerto Rico [25]. Considering that there were no differences in *H. pylori* seroprevalence among men and women in Puerto Rico [11], we could speculate that more symptomatic women sought health care and underwent *H. pylori* eradication treatment. An increasing trend in *H. pylori*-positive GIM cases was observed according to age. This is not surprising because in industrialized countries, including Puerto Rico, the prevalence of *H. pylori* infection increases with age [11, 26].

Our analyses were based on the evaluation of referral-based pathologies received by one private laboratory that receives endoscopic samples from over 50% of all practicing gastroenterologists in Puerto Rico, which could represent a limitation in our study because the data presented may not be representative of the population in Puerto Rico. The lack of information regarding histology and extension of GIM limited our capability to examine the prevalence of GIM subtypes associated with higher gastric cancer risk and highlights the need for organized efforts to disseminate and implement existing biopsy sampling guidelines [21–23] in Puerto Rico. Although some evidence supports that GIM may not give rise to cancer progression, rather, the accumulation of genetic and epigenetic alterations in gastric stem cells in a background of gastric mucosal atrophy leads to the development of gastric tumors [27, 28]; correlations between the prevalence of GIM and regional incidence of gastric cancer have been reported [29, 30]. The significance of our study lies in reporting the baseline epidemiologic data for GIM among Puerto Rican Hispanics, a population with high gastric cancer mortality [2]. Our results emphasize the importance of promoting the implementation of standardized screening and surveillance protocols in Puerto Rico based on the recently published guidelines to improve pathologic diagnosis of GIM, risk stratification, and appropriate surveillance. Future research efforts are needed to identify risk factors and molecular biomarkers associated with GIM among Puerto Rican Hispanics, in order to guide future gastric cancer prevention and surveillance efforts to reduce the disparate gastric cancer burden in this Hispanic subpopulation.

## 5. Conclusions

GIM is considered the principal preneoplastic lesion in the gastric carcinogenesis pathway. This study provides the initial epidemiologic data for GIM among Puerto Rican Hispanics, a population with high gastric cancer mortality. In Puerto Rico, the prevalence of GIM was 10.7% during the study period; 26.9% of the cases were *H. pylori* positive. Most pathology reports lacked information regarding high-risk histologic subtypes and extension. Implementation of the recently published guidelines is necessary to improve pathologic diagnosis and surveillance. To guide gastric cancer prevention efforts in this high-risk Hispanic subpopulation, additional research is warranted to identify risk factors and biomarkers for GIM and to evaluate gastritis staging [31–33] to predict the risk for development of gastric neoplasia in Puerto Rican Hispanics.

## Figures and Tables

**Table 1:** Demographic and clinicopathological characteristics of patients diagnosed with GIM during 2012-2014 (*n* = 4,707).

Characteristic	*n* (%)
Sex	
Male	1,833 (38.9)
Female	2,874 (61.1)
Age	
21-29	46 (1.0)
30-39	120 (2.6)
40-49	290 (6.2)
50-64	1,339 (28.4)
>65	2,912 (61.8)
Mean ± SD	66.0 ± 12.3
Histological type	
Complete	0 (0)
Incomplete	21 (0.4)
Unspecified	4,686 (99.6)
Extension	
Extensive	1,199 (25.5)
Limited	155 (3.3)
Unspecified	3,353 (71.2)

**Table 2:** Biopsy location distribution during the study period (2012-2014).

Location	Total biopsies *n* (%)	Biopsies with GIM *n* (%)	Biopsies without GIM *n* (%)
Incisura angularis	110 (1.5)	102 (92.7)	8 (7.3)
Antrum	4,398 (60.6)	4,016 (91.3)	382 (8.7)
Body	2,370 (32.7)	1,513 (63.8)	857 (36.2)
Fundus	87 (1.2)	45 (51.7)	42 (48.3)
Cardia	44 (0.6)	20 (45.5)	24 (54.5)
Unspecified	244 (3.4)	198 (81.1)	46 (18.9)
Total	7,253	5,894	1,359

**Table 3:** Prevalence of *H. pylori* infections among subjects with GIM according to sex and age during 2012-2014 (*n* = 4,701).

Characteristic	Prevalence (95% CI)
Overall	26.9 (25.7–28.2)
Sex	
Male	31.6 (29.5–33.8)
Female	24.0 (22.4–25.6)
Age group (in years)	
21-29	8.7 (3.3–21.2)
30-39	16.7 (11.0–24.5)
40-49	26.9 (22.1-32.3)
50-64	30.3 (27.9-32.9)
>65	26.1 (24.5-27.7)

## Data Availability

The database, including all of the data in the pathology reports used to support the findings of this study, are available from the corresponding author upon request.
